# Assessing mechanical integrity of spinal fusion by *in situ *endochondral osteoinduction in the murine model

**DOI:** 10.1186/1749-799X-5-58

**Published:** 2010-08-21

**Authors:** Ashvin K Dewan, Rahul A Dewan, Nathan Calderon, Angie Fuentes, ZaWaunyka Lazard, Alan R Davis, Michael Heggeness, John A Hipp, Elizabeth A Olmsted-Davis

**Affiliations:** 1 Spine Research Lab, Baylor College of Medicine, Houston, TX USA; 2 Center for Gene Therapy, Baylor College of Medicine, Houston, TX USA

## Abstract

**Background:**

Historically, radiographs, micro-computed tomography (micro-CT) exams, palpation and histology have been used to assess fusions in a mouse spine. The objective of this study was to develop a faster, cheaper, reproducible test to directly quantify the mechanical integrity of spinal fusions in mice.

**Methods:**

Fusions were induced in ten mice spine using a previously described technique of in situ endochondral ossification, harvested with soft tissue, and cast in radiolucent alginate material for handling. Using a validated software package and a customized mechanical apparatus that flexed and extended the spinal column, the amount of intervertebral motion between adjacent vertebral discs was determined with static flexed and extended lateral spine radiographs. Micro-CT images of the same were also blindly reviewed for fusion.

**Results:**

Mean intervertebral motion between control, non-fused, spinal vertebral discs was 6.1 ± 0.2° during spine flexion/extension. In fusion samples, adjacent vertebrae with less than 3.5° intervertebral motion had fusions documented by micro-CT inspection.

**Conclusions:**

Measuring the amount of intervertebral rotation between vertebrae during spine flexion/extension is a relatively simple, cheap (<$100), clinically relevant, and fast test for assessing the mechanical success of spinal fusion in mice that compared favorably to the standard, micro-CT.

## Background

Spinal fusion is a common surgical procedure used to manage a variety of disorders. In 2001, over 50% of all inpatient lumbar spine operations, other than those for herniated discs, included a fusion procedure [1]. In 2001, $4.8 billion was spent on spine fusion surgery [1]. In 1992, lumbar fusion accounted for 14% of spending, but by 2003, fusion accounted for almost half of total spending on spine surgery [2].

Currently, the gold standard for spinal fusion involves a bone autograft from the pelvis [3]. This technique has several limitations. Donor site complications and morbidity have been estimated at 8% to 25% [4-7]. Donor site complications include pain, nerve and arterial injury, peritoneal perforation, sacroiliac joint instability, and herniation of abdominal contents through defects in the ilium [8]. Furthermore, the volume of bone extracted from the donor is often insufficient [7,9] and pseudoarthrosis is a common result [10]. Given these shortcomings, recent research has focused on finding effective bone graft substitutes, such as bone morphogenic protein (BMP) based osteoinduction.

The feasibility of new technologies is commonly tested in small animal models first. The number of posterolateral fusion studies involving BMP osteodinduction in rodents has exploded in the last decade [11-26]. Research to assess the effectiveness of these new technologies for promoting fusion is compromised however by the lack of a rapid, economical, validated test to determine if the treatment was successful. The recent validation of the rodent as a mechanical model of the human vertebral disc opens the door to new mechanical tests of the rodent spine that can be used to test efficacy, in addition to feasibility, of emerging spinal fusion strategies [27].

**Figure 1:**
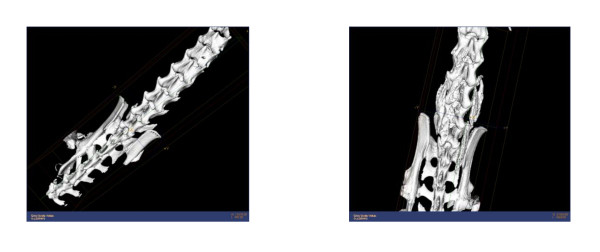
**Spine micro-ct image examples with heterotopic bone formation**.

Historically, radiographs, micro-computed tomography (micro-CT) exams, palpation and histology have been used to assess fusions in a mouse spine. High-resolution micro-CT can reliably determine if a mechanical bridge has formed, but this is expensive, time consuming, and only reliable if the exam is very carefully assessed, since a fusion mass can get very close to a bone but remain separated by a thin layer of soft-tissue (Figure 1). The objective of this study is to develop a rapid and reproducible test to directly quantify the mechanical integrity of spinal fusions in mice. A validated test for fusion efficacy in the mouse spine would be used in many future studies of new biologic fusion technologies.

## Materials and methods

### Cell Culture

Human diploid fetal lung fibroblasts (MRC-5) obtained from American Type Culture Collection (ATCC; Manassas, VA) were transduced with adenovirus encoding BMP-2 as described by Fouletier-Dilling, et al [28]. A control set was also prepared using the same cell line transduced with adenovirus without BMP-2 encoded. For implantation, the control and experimental cells were isolated from the growth medium and re-suspended at 5.6 × 10^6 ^cells/ml in saline medium.

### Implantation

Male and female NOD/SCID mice (8-12 weeks old; Charles River Laoratories; Wilmington, MA) were placed separately at five per cage and fed with an ad libitum diet and tap water in a 12 h day/night cycle according to our Institutional Animal Care and Use Committee (IACUC) protocols until ready for surgery. Experimental protocol was approved by our IACUC. The backs of the mice were shaved and cleansed with alcohol. The senior spinal surgeon listed injected 500ul of the appropriate cell suspension prepared as described above unilaterally adjacent to the spinous process of the L4-L5 vertebrae in mice in the body of the paraspinous muscles in a 1 cm track within the muscle body. Sutures were placed superior and inferior to mark the injection site. The animals were then returned to their respective cage for the remainder of the study.

A total of twenty animals were used for this experiment. Ten mice, 5 female and 5 male, received injections of the experimental cell suspension that produced encoded BMP protein. Ten mice, 5 female and 5 male, received an injection of the control culture that did not encode BMP protein. The mice were euthanized at 6 weeks.

### Mechanical Testing

Following euthanasia, spines were harvested from the first lumbar to the first sacral vertebrae with all surrounding musculature and pelvis intact. The harvested spines were fixed and stored in formaldehyde until ready for testing. Of note, it is unclear what effect, if any, fixation has on the mechanical attributes of the tissue. For mechanical testing spines were first cast in the center of a 2 × 1 × 4 cm block of dental Alginate impression material (Henry Schein, INC., Melville, NY). Next, spines were imaged on high resolution Xray in flexion, neutral, and extension using the custom crafted flexion and extension cells described below. The images were then analyzed using computer-assisted methods on Quantitative Motion Analysis (Medical Metrics, Houston, TX) that has been previously validated [29] and used to assess the mechanical integrity of spinal fusions in human patients. The computer-assisted analysis quantified the amount of intervertebral motion within ±0.1 that occurred in flexion and extension. Following the mechanical testing, the spine was imaged at 14 micron resolution using the micro-CT system. From the micro-CT data, three dimensional reconstructions of the vertebrae and any mineralized tissue were made (eXplore MicroView, v. 2.0, GE Healthcare, London, Ontario). A surgeon blindly reviewed the mouse spine CTs for fusions. Accuracy of spine fusion identification by CT was compared to the mechanical testing of the same spines.

### Testing Apparatus

**Figure 2:**
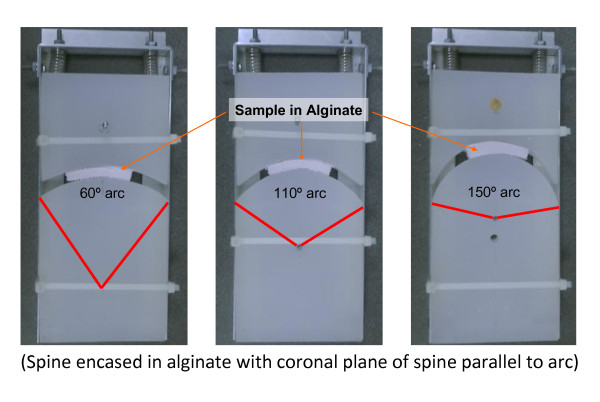
**Custom designed apparatus for flexing/extending explanted spine**.

Three devices were constructed out of radiolucent polyethylene for flexing and extending the mice spines suspended in alginate at 60°, 110°, or 150° (see figure 2). Three 2 × 10 × 20 cm pieces were cut from polyethylene. Using a hack saw and electric sander arcs of 60°, 110°, or 150°, that is arcs with radius of curvature of 10.0, 6.1, and 5.2 cm respectively were cut into the pieces. The arc cuts were made perpendicular to the 10 × 20 cm faces, 10 cm from the top of the long dimension at the edge. A 10 × 23 cm frame to support the plastic pieces was constructed using 2 × 2 cm aluminum L brackets, with the L facing inwards along the longer dimension. Corners of the frame were fastened using separate 1 × 2 × 2 cm L brackets and bolts with nuts. The plastics pieces with the arcs cut into it were next secured to the frame using zip ties. Two 3 cm screws were placed through the frame and polyethylene 2 cm from the bottom edge of the frame to prevent the plastic from sliding out. Two springs 3.75 cm uncompressed length with spring constant of 4.2 N/m were centered on the heads of the two screws supporting the corner L brackets such that an axial force was directed parallel to the long dimension of the plastic pieces.

### Palpation

Integrity of the fusions was qualitatively confirmed after removal of soft tissues with bleach and manual palpation. Sample spines were immersed in 90 cc bleach. After 45 minutes, 6lb fishing line was threaded through the spinal canal of the sample. Samples were then placed into a tray and covered before submerging in bleach again for 2 more hours. Bleach was replaced hourly. Samples with soft tissue remaining on the bones were submerged and monitored for additional 10 minute intervals until bone was completely cleaned. Bones were then photographed using a high resolution camera. Linking of adjacent vertebrae by fusion was documented when present.

### Statistcs

Student's *t*-test was used to compare means of fused and unfused groups. Sensitivity and specificity calculations were performed using Stata Ver 10 (Stata Corp, College Station, Texas).

## Results

**Figure 3:**
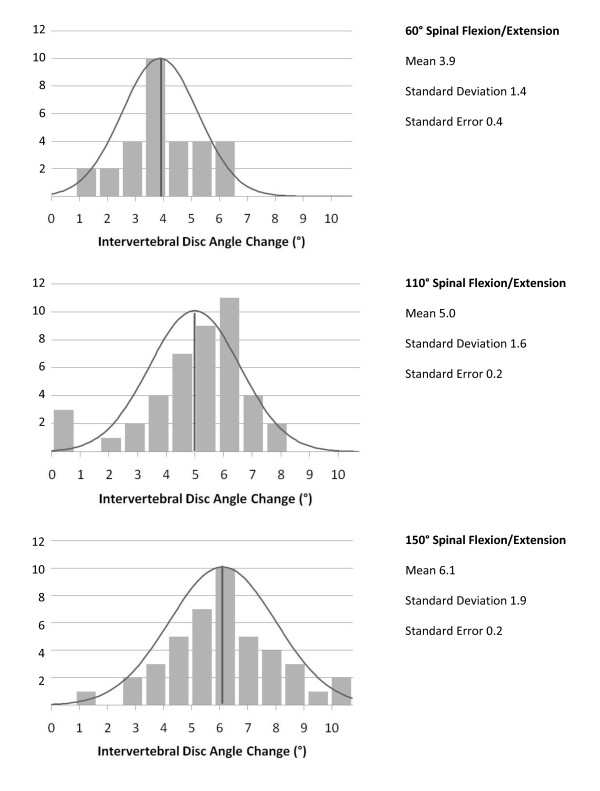
**Histogram of Mean Intervertebral Disc Angle Change in Untreated Mouse Spine during 60°, 110° and 150° of Spinal Flexion/Extension**.

**Figure 4:**
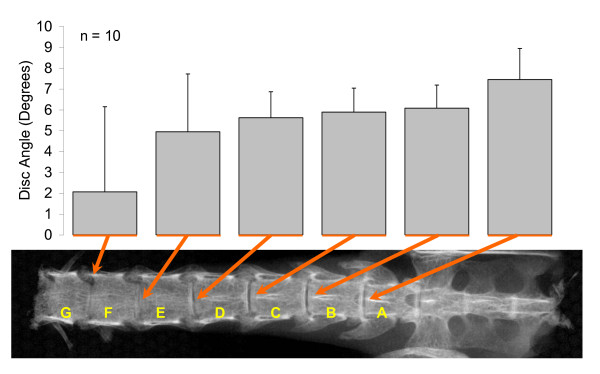
**Mean Intervertebral Disc Angle Change in Untreated Mice Spine at each Vertebral Level during 150 of Spinal Flexion/Extension**.

All mice tolerated surgery without any complications. Biomechanical characterization of untreated control spines was performed first to determine optimal spinal flexion/extension conditions for testing fusion integrity. Maximal intervertebral motion of untreated spines was observed at 150°of spinal flexion/extension. Intervertebral disc angle change of untreated mice followed normal distributions centered at means of 3.9 ± 0.4°, 5.0 ± 0.2°, and 6.1 ± 0.2° per level for 60°, 110°, and 150° of spinal flexion/extension respectively (Figure 3). The greatest variability in intervertebral motion was observed between the proximal lumbar discs of the harvested spine. In addition, mean intervertebral motion between distal lumbar vertebrae levels was slightly greater than mean intervertebral motion at proximal lumbar vertebrae levels (Figure 4), but not significant. Given the small magnitude of intervertebral motion observed at 60° flexion/extension of the untreated spines, subsequent fusion sample testing was conducted successively at only 110° and then 150° for maximal intervertebral disc angle change detection.

Injections of cells producing BMP-2 in the posterior paraspinal muscles resulted in situ endochondral ossification adjacent to vertebrae. Mineralized tissue of varying degrees was present by radiographic examination in all treatment animals at 6 weeks postoperatively. Distinguishing between bridged transverse processes and unbridged mineralized tissue was difficult with anterior-posterior and lateral radiographs. Untreated control animals did not demonstrate any osteoinduction by radiographic examination.

Microcomputed Tomography inspection of explanted spines exposed to BMP-2 was performed taking an average 5 hours/spine (including preparation, scanning, and examination). After 6 weeks of treatment, posterolateral osteoinduction bridging transverse processes of adjacent lumbar vertebral levels were observed in 9/10 treated spines. Fusion occurred at greater than two adjacent vertebrae for 5 of these spines. One such spine had 5 successive lumbar vertebrae, L1-L5, fused. The only spine that did not produce any fusion by micro-CT had a small amount of bone formation localized in the paraspinal muscle.

**Figure 5:**
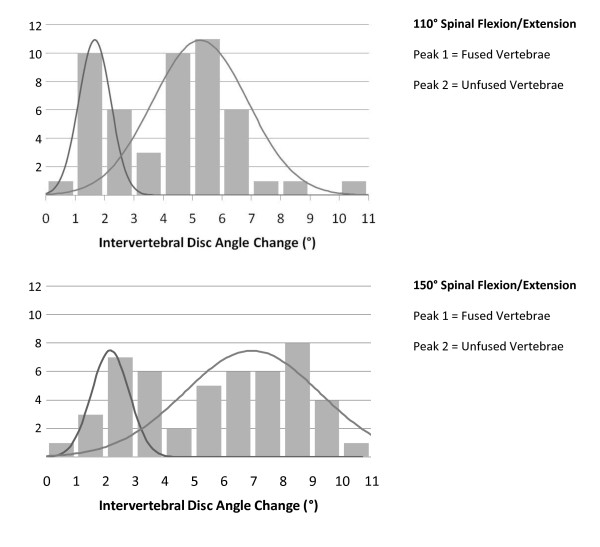
**Histograms of Mean Intervertebral Disc Angle Change During 110° and 150° of Spinal Flexion/Extension After Six Weeks Exposure to Bone Morphogenic Protein-2**.

Biomechanical characterization of treated spines was performed at 110° and then 150° spinal flexion/extension. The intervertebral motion between lumbar discs neighboring the mineralized tissue masses decreased. A compensatory increase in intervertebral motion between lumbar discs away from the mineralized tissue was observed at both 110° and 150° testing. Two separate peaks of intervertebral disc angle change representing the linked and unlinked vertebrae from the pool of all the treated vertebrae were observed at both testing conditions (Figure 5). Mechanical data of fusions were correlated with CT findings next. Restriction of intervertebral motion by mineralized tissue neighboring the spine was variable. However, it was noted, with the exception of two unfused adjacent vertebrae, all other adjacent vertebrae that lacked fusion by CT inspection exhibited greater than 3.5 degrees of intervertebral motion with the 150 degree flexion/extension testing condition.

Soft tissue envelopes of explanted spines were successfully dissolved using bleach. Segments of fused vertebrae in treated spines were palpated to confirm mechanical integrity. After 6 weeks of exposure to BMP-2, all 10 spines grossly exhibited linked vertebrae. Furthermore, 8 of these spines had greater than 2 adjacent linked vertebrae, with one spine exhibiting fusion from L1-L5 after bleach dissolution.

**Figure 6:**
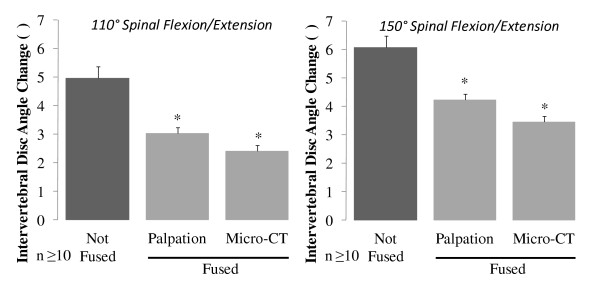
**Comparison of Mean Intervertebral Disc Angle Change during Spinal Flexion/Extension of Bone Morphogenic Protein-2 Induced Spinal Fusions Identified by Palpation and Micro-CT Techniques**.

Levels coded as fused by palpation after BMP-2 exposure showed significantly decreased (p < 0.05) intervertebral motion at 110° and 150° testing (2.4 ± 0.3° and 4.2 ± 0.4° respectively) compared to controls (Figure 6). Levels coded as fused by micro-CT after BMP-2 exposure also showed a significant decrease in intervertebral motion at 110° and 150° testing (3.1 ± 0.3° and 3.5 ± 0.4° respectively) compared to controls. Fusions identified by micro-CT however were relatively more stable compared to the fusions found by palpation. The lower rate of false positive fusions by the micro-CT relative to the palpation group might explain the decreased intervertebral motion observed. For both methods of identification, the percentage of intervertebral motion decrease from fusion was greater at 110° testing compared to 150° testing.

Finally, the sensitivity and specificity of mechanical testing of fusion was calculated. The challenge in performing these statistics was the lack of a definitive gold standard. Our perception is that a very careful assessment of micro-CT exams is the best method, but none of the assessments made can be assumed to be correct 100% of the time. Using micro-CT assessment as the gold standard, 84% of the levels analyzed were correctly classified using our mechanical test. The sensitivity and specificity for identifying a fusion that limited intervertebral motion to ≤3.5° under the 150° mechanical testing condition was 54% and 94% respectively. Compared to micro-CT, there were false-negative assessments by mechanical testing. Or stated another way, fusion masses qualitatively identified on Micro-CT as bridging or fusing adjacent vertebrae, did not necessarily restrict the intervertebral motion.

## Discussion

This is the first study to characterize the rodent spine in flexion-extension testing. Incorporating the same methodology used in human spine testing, we were able to assess spinal fusion in the rodent model. In humans, quality of spinal fusions is typically assessed through dynamic and static imaging studies [10,29]. After performing spinal fusion, surgeons take radiographs of a patient's spine in flexion and extension. Based on the limitations in motion observed between two vertebrae after fusion, a surgeon can assess the quality of the fusion. Lately, software has become available that quantifies the degree of intervertebral motion between vertebral discs [29]. Using the same software and a simple, custom-designed, apparatus (Figure 2) to flex and extend the explanted rodent spines for radiographs, we were able to reliably measure interverteral motion in the rodent lumbar spine.

Currently the most common methods for fusion assessment in the rodent model include histology, palpation, micro-computed tomography, and radiography. All of these techniques are qualitative with noteworthy limitations. Histology is accurate at evaluating bone formation and quality, but it is easy to miss bridging bone in out of plane sections when looking for fusions [16,25]. Moreover static images of individual sections do not reveal how the newly mineralized tissue functions during physiologic motion of the spine. Palpation of interlocked segments is used to classify motion segments as fused or not fused. Although relative determinations of fusion strength can be made, this admittedly subjective technique [26] suffers from significant interobserver variation and unclear relevance to the clinical setting. Nonetheless, there are some authors that believe palpation is the most sensitive and specific method of assessing spinal fusion [18,25,30]. Most consider micro-CT to be the gold standard for fusion determination [16]. On micro-CT bony bridging between adjacent transverse processes is considered fusion. CT is time consuming (5 hours/sample in this study) and expensive. Moreover, determining the significance in the variability of fusions observed can be challenging. Consequently, some consider the combination of micro-CT and palpation to be optimal [16]. The success rates of fusion induced by BMP-2 determined by micro-CT and/or palpation reported in literature are 95-100% [11,12,14,17-19,21,22,24], consistent with our micro-CT and palpation findings. Finally some studies use radiographic evidence of bony tissue along the margin of the spine to assess fusion. This is perhaps the most misleading however since adjacent and integrated mineralized tissue cannot be readily distinguished leading to overestimation of fusion [16]. There is no consensus about which technique is best for assessing fusion.

Given limitations of current techniques for spinal fusion assessment, we developed a quantitative biomechanical test of intervertebral motion in the rodent spine. Untreated lumbar mice spines behaved very similar to untreated human and rabbit lumbar spine described in literature [29,30]. Mean intevertebral motion at L3-L5 of 5.7° reported during flexion and extension of the human spine is very similar to the mean intervertebral motion of 6.1° demonstrated in flexion and extension of the mouse spine here [29]. Consistent with trends demonstrated in human and rabbit lumbar vertebrae, higher rodent lumbar levels also showed slightly less intervertebral motion compared to the lower lumber levels [31].

Defining normal intervertebral motion enabled us to objectively assess the fused rodent spines. The cut-off that correlated with fusion by micro CT we used, 3.5 degrees, was within the 2°-4° range of cut-offs reported for fusion in other models [32,33]. Characterization of fusion products revealed a great deal of variability in the quality of fusions, not detected by the existing fusion detection techniques. The induction of bone at a heterotopic site in the mouse did not necessarily imply the induction of directed formation of bone essential for spinal arthrodesis [10]. Often heterotopic bone bridging transverse processes of the vertebrae was not capable of restricting intervertebral motion during spinal flexion/extension. In our testing, 6/16 vertebral fusions identified by micro-CT were not able to restrict intervertebral motion less than 3.5 degrees. These 6 "false" negatives result in a lower sensitivity of mechanical testing when compared to micro-CT, the defacto standard. However, using the quantitative mechanical technique to assess fusions permited the identification of these pseudoarthroses, and provided additional objective information about the quality of the fusions generated.

Grauer et al similarly identified differences in fusion quality not detected by palpation in flexion-extension testing of a rabbit model [30]. In their experiment, with the absence of a carrier for injected induction proteins, the location of bony fusion masses induced was not precise. The variability in fused domains could explain the variability in intervertebral motion observed. With palpation alone, the significance of fusion domains was harder to appreciate. In cadavers, Bono et al demonstrated the same concept, noting intertranverse process bridging reduced inervertebral motion less than interspinous processes bridging [32].

A few authors have attempted to devise other quantitative biomechanical tests for assessing the integrity of spinal fusions in small animal models. Most of these published tests however require sophisticated equipment. In rabbits, uniaxial tensile mechanical testing of fusions has been performed [34]. The smaller scale of rodent model fusions however makes this technique prohibitive and tedious. Grauer et al developed a flexibility test for intervertebral motion in the rabbit [31]. Another group has compared displacement of fused rat spine in the sagittal plane with the application of a 3N force [13]. Generalizing the observations of these ex vivo tests to the clinical setting however can be trickier given that the same approaches are not used in the human.

Finally, the cost of test described here is another advantage. A dedicated microcomputed tomorgraphy machines with enough resolution to accurately image mice spines is usually not readily available. At our institution, multiple labs share this resource. A single machine can cost upwards of $100,000 and requires routine costly maintenance. In contrast, the test shown here can be performed on a rudimentary high resolution Xray machine that many institutions already have. Laboratory x-ray systems can cost between $5,000 to $50,000 depending on the system and whether it is purchased new or used. The software that was used in this study is not yet available for purchase in a stand-alone laboratory setting. Other computer-assisted methods have been described that would likely have similar accuracy for this purpose [35,36]. Some spine centers may already have such software for the analysis of human spinal motion. The cost of constructing the actual testing apparatus was less than $100.

## Conclusion

Measuring the amount of intervertebral rotation between vertebrae that occurs during flexion and extension is a relatively simple, cheap (<$100), clinically relevant and fast test for assessing the mechanical success of spinal fusion in mice. Existing methods of spinal fusion assessment such as micro-computed tomography (micro-CT) are time-consuming and cost prohibitive. Quantitative analysis of intervertebral rotation between flexion and extension can be used to reliably determine if adjacent vertebrae are fused, with fused levels having less than 3.5 degrees of intervertebral rotation during 150 degrees of spinal flexion/extension. The recent validation of the rodent as a mechanical model of the human vertebral disc opens the door to new mechanical tests of the rodent spine that can be used to test efficacy, in addition to feasibility, of emerging spinal fusion strategies [27]. With the explosion in the number of studies using the rodent model for posterolateral spinal arthrodesis in the last few years [11-26], the development of a rapid, reproducible, biomechanical test for fusion assessment in rodents, such as the one described here, is essential.

## Abbreviations

BMP: Bone Morphogenic Protein; Micro-CT: Micro-Computed Tomography.

## Competing interests

J. Hipp is founder of Medical Metrics, INC., developer of the Quantitative Motion Analysis Software Package used here.

No other competing interests to declare.

## Authors' contributions

AKD drafted manuscript, constructed mechanical testing apparatus, designed testing protocols, and analyzed final data. RAD prepared and tested spine samples and helped with computer analysis. NC helped with construction of testing apparatus and computer analysis. AF helped with sample preparation and Micro-CT testing. ZL helped prepare viral vector with BMP and fibroblasts for surgical injection. ARD provided lab resources, necessary cell lines, and guidance for viral vector preparation. MH performed surgical exposures and injections and participated in design and coordination. JAH conceived of study, and participated in design and coordination. EAO provided lab animal resources and equipment for tests, and participated in design and coordination. All authors read and approved the final manuscript.
